# The Heart during Convalescence from Febrile Disorders

**Published:** 1910-05-21

**Authors:** 


### THE HEART DURING CONVALESCENCE FROM FEBRILE DISORDERS.

In many febrile conditions endocarditis and pericarditis may be absent, and yet cloudy swelling and fatty degeneration both of the heart muscle and of the nerve fibres may be the actual cause of death. The muscle fibres may be friable, the heart flaccid, and the walls thin, even though there may be no obvious dilatation. The muscle is usually soft and of a reddish-brown or even of a yellowish-brown colour. It is exceedingly unlikely that these changes occur only in the heart of a patient who is going to die; minor degrees of similar conditions almost certainly exist in patients who recover, and they are probably present not only during the height of the fever but for a variable period after the acute stages of the disease are past and the patient has become convalescent.

### The Heart Muscle in Febrile Conditions.

This view about the heart fibres in fevers is not kept clearly enough in mind as a rule, and in a few cases that which is sometimes spoken of as functional disorder of the heart after acute febrile illnesses is doubtless really due to the patient having been allowed to return to his ordinary duties when the heart muscle had not yet entirely recovered from its cloudy swelling. This is especially seen, perhaps, after typhoid fever, but it is also obvious even after fevers of short duration, especially true influenza; the difference between influenza and pneumonia in this respect is remarkable, for it is almost astounding how rapidly the majority of patients recuperate after a severe attack of the latter.

### Some Practical Points.

The questions that arise are: When is it wise to allow a patient after an acute fever to sit up? When should he be permitted to get up? How soon should he be permitted to take exercise? And for the sake of safety it is clear that it will often save time to hasten slowly. Those who have considered the question believe that after typhoid fever it is as a rule unwise for the patient even to sit up in bed until a week or even 10 days have elapsed from the time that the temperature has begun to remain normal. He should not leave his bed until he has tried sitting up in bed several times for increasing periods, and even then not until it has been shown that the sitting up in bed is not markedly increasing the rapidity of the heart's action or the pulse rate. If sitting up in bed causes a weakness of the first cardiac sound, the patient is safer not to sit up yet for a few days, whilst if sitting up in bed causes a rapid action of the heart or, still worse, either intermittency or irregularity, great caution should be employed throughout convalescence. Other indications that there has been considerable cloudy swelling of the heart muscle during a fever are the occurrence of a blurring of the first sound of the heart, amounting sometimes to a systolic bruit at the cardiac impulse when the patient begins to exert himself; marked differences between the blood-pressures taken by some suitable recording instrument, and not merely guessed at by palpation of the pulse, whilst the patient is lying flat upon his back, upon the one hand, or sitting upright, upon the other; marked subnormality of the temperature; a tendency to lessening of the amount of urine passed when the patient has begun to exert himself; or marked slowing of the pulse under similar circumstances. There are certain patients who, owing to the fact that too much work was demanded of the heart at the time when its protoplasm was in a state of partial cloudy swelling, suffer from attacks of faintness or of other indications of cardiac dilatation for weeks, months, or even years afterwards.

### The Question of Prognosis.

Naturally, the rate of recovery of the heart muscle will be greater in those who have previously been most healthy, and in the young rather than the old. If the patient is young and has hitherto been well, the prognosis as regards the heart is good even although the infectious disease has been of a severe type, whereas in patients near or past middle life much more care needs to be exercised. The point to be remembered is that although there may be no obviously abnormal physical signs about the heart at all, there is bound to be a certain amount of cloudy swelling of its protoplasm in every infective fever, and that it is by forgetting this pathological point that too hasty an augmentation of the exertion permitted during convalescence may lead to long-standing or even permanent cardiac symptoms afterwards.

